# Injectable exosome-functionalized extracellular matrix hydrogel for metabolism balance and pyroptosis regulation in intervertebral disc degeneration

**DOI:** 10.1186/s12951-021-00991-5

**Published:** 2021-09-06

**Authors:** Hongyuan Xing, Zengjie Zhang, Qijiang Mao, Chenggui Wang, Youlong Zhou, Xiaopeng Zhou, Liwei Ying, Haibin Xu, Shaojun Hu, Ning Zhang

**Affiliations:** 1 Department of Orthopedics Surgery, 2nd Affiliated Hospital, School of Medicine, Zhejiang University, Hangzhou, 310009 Zhejiang People’s Republic of China; 2 Department of General Surgery, Sir Run Run Shaw Hospital, School of Medicine, Zhejiang University, Hangzhou, 310028 China; 3 Department of Orthopedics, Changxing People’s Hospital, Changxing, China

**Keywords:** Intervertebral disc degeneration, Exosome, Extracellular matrix, Pyroptosis, Tissue engineering

## Abstract

Exosome therapy is a promising therapeutic approach for intervertebral disc degeneration (IVDD) and achieves its therapeutic effects by regulating metabolic disorders, the microenvironment and cell homeostasis with the sustained release of microRNAs, proteins, and transcription factors. However, the rapid clearance and disruption of exosomes are the two major challenges for the application of exosome therapy in IVDD. Herein, a thermosensitive acellular extracellular matrix (ECM) hydrogel coupled with adipose-derived mesenchymal stem cell (ADSC) exosomes (dECM@exo) that inherits the superior properties of nucleus pulposus tissue and ADSCs was fabricated to ameliorate IVDD. This thermosensitive dECM@exo hydrogel system can provide not only in situ gelation to replenish ECM leakage in nucleus pulposus cells (NPCs) but also an environment for the growth of NPCs. In addition, sustained release of ADSC-derived exosomes from this system regulates matrix synthesis and degradation by regulating matrix metalloproteinases (MMPs) and inhibits pyroptosis by mitigating the inflammatory response in vitro. Animal results demonstrated that the dECM@exo hydrogel system maintained early IVD microenvironment homeostasis and ameliorated IVDD. This functional system can serve as a powerful platform for IVD drug delivery and biotherapy and an alternative therapy for IVDD.

**Supplementary Information:**

The online version contains supplementary material available at 10.1186/s12951-021-00991-5.

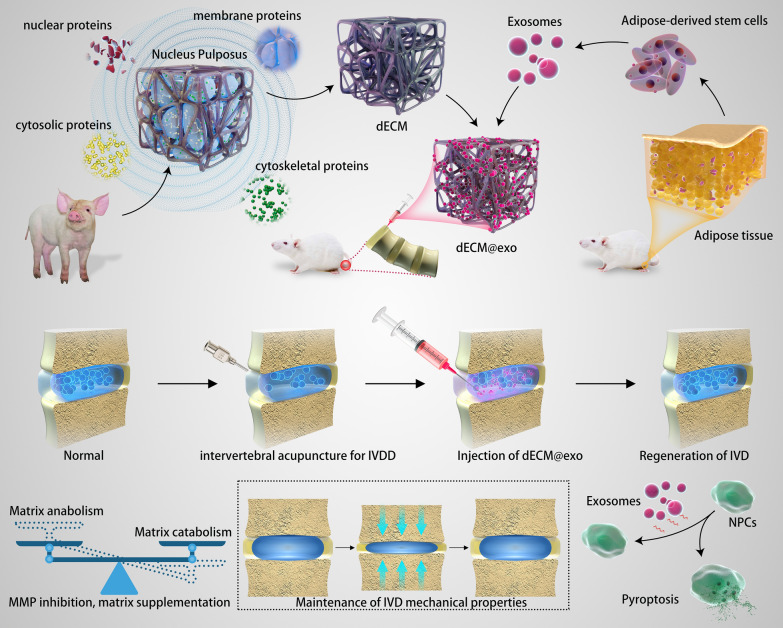

## Introduction

Intervertebral disc degeneration (IVDD) is a chronic degenerative disease that consumes a substantial amount of medical resources [1–3]. The main mechanism of IVDD are the disorder of catabolism and anabolism in the extracellular matrix (ECM) and changes in the intervertebral disc (IVD) microenvironment [4]. During IVDD, the abnormal expression of matrix metalloproteinases (MMPs) and reduced collagen II decreased the secretion of type II collagen from the nucleus pulposus (NP), resulting in the disruption of the ECM balance [5, 6]. IVD microenvironment remodeling and the aggregation of inflammatory factors as well as the death of NPCs result in a cascade of worsening reactions [7–9]. Therefore, restoring the IVD microenvironment and protecting nucleus pulposus cells can ameliorate IVDD.

Mesenchymal stem cell (MSC) transplantation, as a representative cell therapy, is becoming prevalent in the field of fracture healing [10], cartilage repair [11, 12], spinal cord injury repair [13] and intervertebral disc regeneration [14]. However, stem cell transplantation has not been widely applied due to defects in tumorigenesis and immune rejection. Paracrine signaling is the main means of cellular communication, and exosome secretion is a common form of paracrine modulation. By inheriting the properties of MSCs, extracellular vesicles (EVs) protect NPCs from apoptosis, promote the synthesis of ECM, and mitigate the inflammatory response in discs [15]. Exosomes are EVs that play a major role in paracrine signaling and have the potential to achieve therapeutic efficacy similar to MSC transplantation in the treatment of IVDD. Exosomes contain a large number amounts of proteins, RNA, DNA, amino acids and metabolic molecules. Furthermore, there are also many functional proteins on the exosome surface that can play unique roles. Adipose-derived mesenchymal stem cell (ADSC) exosomes have been confirmed to promote the proliferation and inhibit the apoptosis of target cells and have anti-inflammatory effects in the treatment of tendon lacerations [16, 17]. In addition, exosomes have been demonstrated to be capable of affecting ECM catabolism by inhibiting MMPs [18, 19]. Therefore, exosome therapy is considered an ideal alternative to cell therapy for IVDD.

While exosome therapy has the advantages of low immunogenicity and suitability for the IVD microenvironment, some problems remain to be solved [20]. Since the intervertebral disc is a closed, avascular structure, intervertebral injection is an ideal method for the treatment of IVDD [21]. However, the interaction time for simple exosome injection is too short to achieve long-term effects, and repeated injection in clinical treatment is obviously infeasible in clinical treatment. To solve these problems, an exosome carrier suitable for the IVD is needed. Traditional exosome carriers have some problems, such as cytotoxicity, low biocompatibility and difficult degradation [22]. As an ideal carrier, ECM acellular biological scaffolds with good biocompatibility and low cytotoxicity have been widely applied for degenerated discords such as osteoarthritis and IVDD [23–25]. In addition, the immunogenicity of the ECM can be reduced by a decellularization process, and exosomes can easily adhere to the fibers of the ECM.

**Scheme 1:**
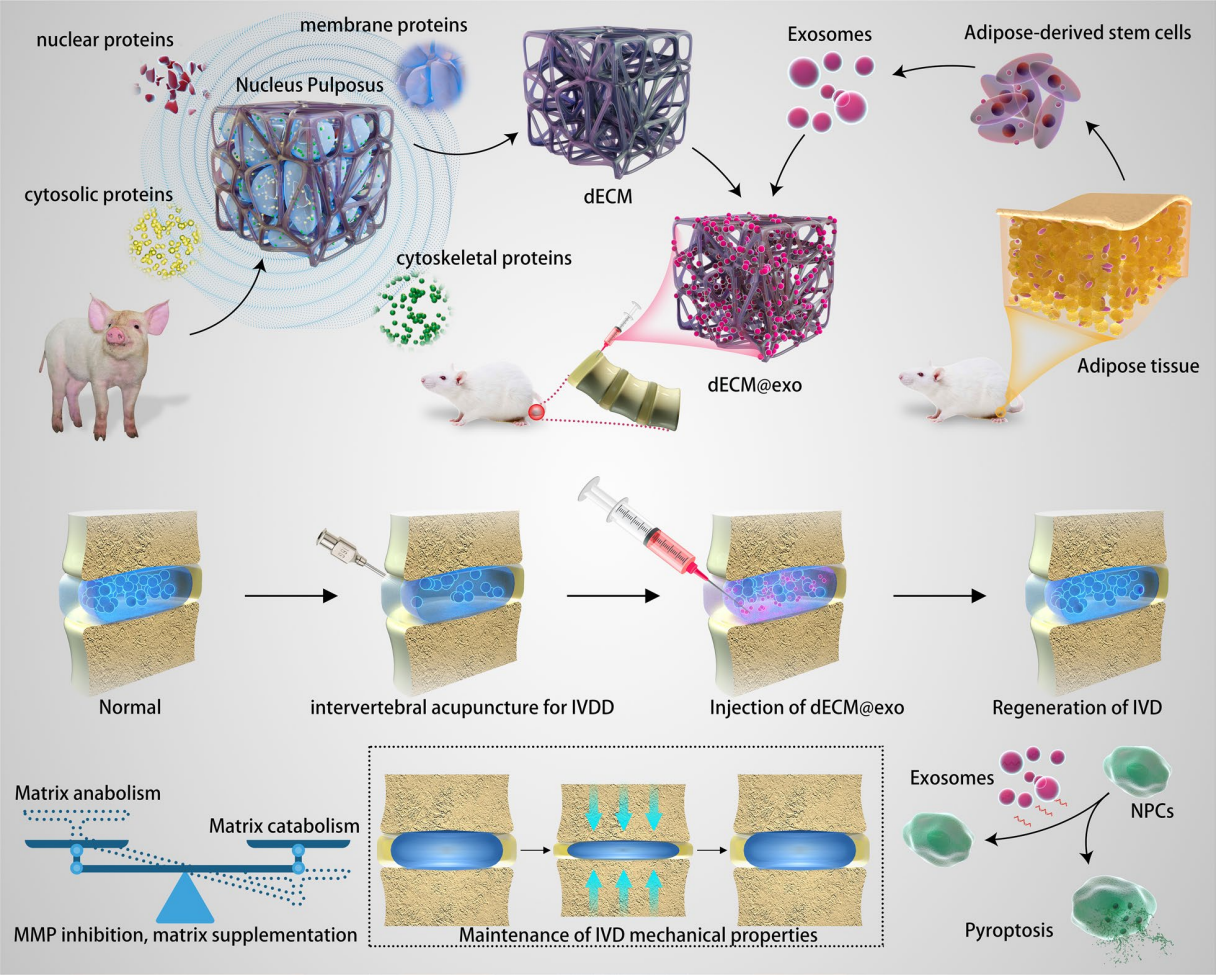
dECM@exo synthesis and its mechanisms in the treatment of intervertebral disc degeneration

In this study, taking advantage of the microenvironment regulation function of exosomes, the rapid in situ gelation ability of ECM hydrogels at body temperature and the good biocompatibility of ECM hydrogels, an injectable thermosensitive hydrogel system was constructed via coordinative crossing of ADSC-derived exosomes and ECM hydrogels to effectively restore the microenvironment and protect NPCs from pyroptosis after IVDD (Fig. 3). In this paper, a new method of preparing ECM hydrogels for the treatment of IVDD is proposed. Compared with traditional scaffolds, the ECM hydrogels do not contain toxic crosslinking agents, and the properties of the ECM hydrogels are more similar to those of NP tissue. In this study, exosomes derived from adipose-derived mesenchymal stem cells (ADSCs) were harvested and immobilized on ECM hydrogels. The ECM biological scaffolds loaded with exosomes (dECM@exo) were proven to be injectable and temperature sensitive in vitro. In addition, we investigated the effects of dECM@exo on matrix catabolism, pyroptosis, and inflammation in degenerative NPCs. Moreover, using a rat model of tail vertebral disc degeneration, dECM@exo was confirmed to alleviate the degradation of ECM and promote ECM regeneration (Scheme 1). Herein, we aimed to develop a novel IVD biological hydrogel to regulate the microenvironment, treat IVDD and provide a new vector for exosomes.

## Materials and methods

### Scaffold fabrication

Fresh porcine NP tissue was carefully dissected and obtained from the spine. The NP tissue underwent five freeze-thaw cycles before the enrichment process. Basic reagents for the enrichment of ECM were selected based on previous research [26]. A mixture of buffers (5 ml of each buffer C and buffer W) was added to 500 mg of NP tissue and homogenized in a 4 °C shaker for 1 h. The obtained homogenate was centrifuged at 16,000*g* for half an hour, and the underlying mixture was collected. Three milliliters of buffer N that had been added to deoxyribonuclease I (400 µg/ml, D5319, Sigma, Shanghai, China) and ribonuclease A (20 µg/ml, R6513, Sigma, China) was added to the homogenate. The homogenate was shaken in a 4 °C shaker for an hour and then centrifuged at 16,000*g* for half an hour, and then the supernatant was removed. Buffer N was again added, and this step was repeated three times. The obtained homogenate was put into 4 ml of buffer M, mixed in a 4 °C shaker for 40 min, centrifuged at 16,000*g* for half an hour, and then the supernatant was removed. The obtained homogenate was put into 1 ml of CS buffer, mixed on a room temperature (RT) shaker for an hour, and centrifuged at 16,000*g* for half an hour to remove the supernatant. The product was added to 1 ml of buffer C, mixed in a 4 °C shaker, and centrifuged at 16,000*g* for 1 h. Peracetic acid (0.01%) was used for sterilization. Finally, 5 ml of phosphate-buffered saline (PBS) was added to wash the product, followed by centrifugation at 16,000*g* for 10 min three times.

### Characterization of ECM in dECM samples

The samples of fresh NP and dECM were fixed in 4% paraformaldehyde and dehydrated with 30% sucrose for 3 days. Section (8 μm) were cut with a freezing microtome (M630, Medite GmbH). A 1 µg/ml 4′,6-diamidino-2-phenylindole (DAPI; Sigma, China) solution was used to stain the frozen slides for half an hour in the dark after rinsing with PBS. A fluorescence microscope (DMi8; Leica) was used to observe the slides after three washes with PBS. The amount of DNA in the sample was tested by a Genomic DNA Kit (TIANGEN, Beijing, China). A microplate spectrophotometer (Thermo Fisher Scientific, USA) was used to determine the DNA concentration from the absorbance at 260 nm. Hematoxylin and eosin (H&E) was used to observe the distribution of cells. Alcian blue staining and Sirius Red staining were performed to determine the proteoglycan and collagen distribution. Collagen content was quantitatively determined using a Total Collagen Assay (ab222942, Abcam).

### Isolation and identification of exosomes

Sprague-Dawley (SD) rat ADSCs were purchased from Cyagen Bioscience (Guangzhou, China). The corresponding growth medium (Saiye Biotechnology Co.) was used to culture the ADSCs. Exosomes were extracted and purified from ADSCs as previously described [27]. Briefly, ADSC culture medium was collected every day and centrifuged at 300*g* for 10 min, followed by an additional 10 min at 2000*g* and then at 10,000*g* for half an hour to remove the lifted cells and cell debris. Then, the supernatant was collected in an ultrafiltration tube (UFC9010, Millipore, Shanghai, China) and centrifuged at 3500*g* for 10 min, and the concentrated solution from the upper tube was collected for filtration through a 0.2-µm pore membrane filter. The supernatant was ultracentrifuged at 100,000*g* for 70 min at 4 °C using a 70Ti rotor (Beckman Coulter). Then, the liquid in the centrifuge tube was carefully removed while an equal amount of PBS was added. Finally, the supernatant was removed by centrifugation at 10,000*g* for 70 min, and 5 µl of PBS was used to collect the exosomes. The exosomes obtained were stored at − 80 °C. A Micro BCA protein assay kit (BOSTER, Wuhan, China) was used to quantify the exosomes. The proteins Alix, TSG101 and calnexin, which are related to exosomes, were analyzed using Western blotting (WB) with primary antibodies as follows: anti-Alix (ab186429, 1:1000, Abcam), anti-TSG101 (ab125011, 1:1000, Abcam), and anti-calnexin (ab133615, 1:1000, Abcam) according to a previous study. A ZetaView PMX 110 (Particle Metrix, Germany) was used for the NTA (nanometer size) analysis of the exosome samples. ZetaView 8.04.02 SP2 was used to analyze the results. Exosome samples were observed using a JEM-1400 instrument (JEOL, Japan).

### Physicochemical and multifunctional property characterizations of dECM@exo

dECM@exo was obtained by mixing 1 µg of exosomes with 100 µL of dECM with stirring at 4 °C. Both dECM and dECM@exo were characterized by Fourier transform infrared (FTIR) spectrophotometry (Nicolet iS 10). Spectra were recorded in transmission mode with 32 scans from 4000 to 400 cm^−1^. Scanning electron microscopy (SEM) (GeminiSEM 300, ZEISS) was used to observe the porous morphology of the dECM@exo. The rheological properties of the scaffolds, including the storage modulus (G′) and loss modulus (G′′), were determined by employing a rheometer (HAAKE MARS60, Germany). The temperature effects on dECM@exo ranging from 4 to 50 °C were evaluated by maintaining the strain and frequency constant at 1% and 1 Hz, respectively. The injectability of the biological scaffolds was measured by the change in viscosity with the shear rate.

### Profile of exosome release from the dECM@exo hydrogels

The exosome release profile was determined with a Rat CD63 ELISA Kit (MEIMIAN, Shanghai, China). Briefly, 100 µl of the above prepared dECM@exo containing 1 µg of exosomes or 100 µl of dECM@exo alone were placed in the upper chamber of a Transwell insert (Corning, China) chamber in a 24-well plate, and 200 µl of PBS was added to the lower chamber. Subsequently, 100 µl of PBS (pH = 5.5) was collected and replaced with 100 µl of fresh PBS on days 0, 4, 8, 12, 16, 20, 24 and 28. The number of released exosomes was determined, and the exosome release percentage was calculated.

### Cell culture

NPCs were obtained from the nucleus pulposus tissue of 8-week-old SD rats (n = 4). Briefly, NP tissue was carefully removed from the disc. Then, the obtained tissue was cut into paste and digested with 0.1% collagenase (Gibco, Shanghai, China) at 37 °C for 20 min. Dulbecco’s modified Eagle’s medium: F-12 (Gibco) was used to stop digestion, and the mixture was centrifuged at 1000*g* for 5 min. Primary culture was performed using DMEM/F12 in a humidified incubator at 37 °C with 5% CO2. The medium was changed every other day, and passaging was carried out when the cell density reached 80%. The third generation of cells was used in subsequent experiments.

### Cell cytotoxicity and proliferation assessments

The cytotoxicity and proliferation of the cells cultured with dECM and dECM@exo for 1, 3 and 7 days were determined with a CCK-8 assay kit (BOSTER, Wuhan, China). Ten microliters of CCK-8 solution was added to NPCs followed by incubation at 37 °C for 2 h. A Varioskan LUX (Thermo Scientific, USA) was used to measure the absorbance value at 450 nm. A Calcein/PI Cell Viability/Cytotoxicity Assay Kit (Beyotime, China) was used to detect the distribution of living and dead cells. Calcein-AM (1 µM)/propidium iodide (PI) (1 µM) was added to NPCs. NPCs were incubated in a 37 °C incubator for 5 min. Then, a fluorescence microscope (Leica) was used to obtain fluorescence images.

### Immunofluorescence

NPCs were fixed on 24-well plates with 4% paraformaldehyde for 15 min followed by PBS washing three times, and 0.2% Triton X-100 (W/V) in PBS was added for 15 min. The cells were blocked with 5% bovine serum albumin (BSA) for 30 min after three washes with PBS. Primary antibodies against Ki67 (ab1558, 0.5 µg/ml, Abcam, USA), MMP13 (18165-1-AP, 1:100, Proteintech, China), type II collagen (1:100; Novus, Shanghai, China) or polyclonal rabbit anti-GSDM-D (1:100, Proteintech, China) were added followed by incubation at 4 °C for 12 h. Then, the secondary antibody [Alexa Fluor 555-labeled goat anti-rabbit IgG (1:500, Beyotime, China) or goat anti-rabbit Alexa Fluor 488 (1:500, Beyotime, China)] was added for incubation for 1 h. Images were captured with a fluorescence microscope (Leica).

### WB

Proteins were extracted from cell samples by moderate-strength RIPA buffer supplemented with a proteasome inhibitor (BOSTER, China). Then, the proteins were separated using 10% SDS-PAGE and transferred from the gel to a polyvinylidene fluoride membrane (Millipore, Shanghai, China). The membranes were subsequently blocked with 10% skimmed milk for an hour and washed with Tris-buffered saline with 0.1% Tween-20 (TBST) three times. Subsequently, the membranes were incubated with anti-cleaved N-terminal GSDMD (ab215203, Abcam), anti-caspase-1 (ab207802, Abcam, China), anti-NLRP3 (ab263899, Abcam), or anti-IL-1β (ab254360, Abcam, China) for 12 h. The internal control was anti-GAPDH (ab181602, Abcam). After washing away the excess antibodies with TBST, the membranes were incubated with specific horseradish peroxidase-conjugated secondary antibodies (Beyotime, China) at room temperature for one hour. The immunoreactive bands were observed with a ChemiDoc Touch imaging system (Bio-Rad) to measure the signal intensity.

### Animal surgery

SD rats (n = 48, 250–270 g, male) were purchased from Shanghai Slack Laboratory Animal Co., Ltd. (China). The study was approved by the local authorities (Zhejiang Chinese Medical University Laboratory Animal Research Center, China). SD rats were anesthetized with 1% pentobarbital sodium (P-010, Merck, China) at a dose of 4 ml per kilogram. Then, the intervertebral spaces of coccygeal vertebrae (Co) were exposed [28, 29]. The rats were grouped as follows: (1) the NC group (without needle puncture); (2) the DC group (with needle puncture and PBS injection); (3) the dECM group (with needle puncture and injection of dECM); and (4) the dECM@exo group (with needle puncture and injection of dECM@exo). Then, an 18-gauge puncture needle was inserted 5 mm into each disc, rotated 90° clockwise, and held for 45 s. Five microliters of material (or PBS) was injected through a 22-gauge needle into the NP tissue of Co7/Co8, Co8/Co9, and Co9/Co10.

### Disc height measurement

A molybdenum target (MCR-6000, China) was used to obtain X-ray images of the IVDs at 0, 4, 8, and 16 weeks. ImageJ (National Institute of Health, USA) was used to measure the disc height.

### Histological and immunohistochemical analyses of the IVD

The caudal vertebrae of rats were harvested at 0, 4, 8, and 16 weeks. The samples were fixed in 4% paraformaldehyde for 2 days and then decalcified using 10% EDTA (BOSTER, China). After decalcification was complete, the tissues were dehydrated and embedded in paraffin. The paraffin specimen was cut into sections of 5 μm. H&E staining and safranin O–fast green (S–O) staining were used to assess the degree of IVDD in each group. Two blinded observers assessed the cellularity and morphology. The grading method was based on a previously reported method [30]. Collagen ll and Aggrecan were used as immunohistochemical indicators to assess the degree of intervertebral disc degeneration. H_2_O_2_ (3%) was used to treat the samples for 10 min. Then, the samples were blocked with 5% BSA for half an hour at room temperature followed by hybridization with mouse anti-rabbit type II collagen antibody (1:100; Novus, China) or mouse anti-rabbit aggrecan antibody (1:100; Novus) at 4 °C overnight. After washing five times with PBS, a biotin-labeled secondary antibody was used to incubate the samples at 37 °C for 30 min. The SABC method was used to detect staining.

### Statistical analysis

All experiments were performed on at least three individual samples. A value of *P < 0.05 was considered a significant difference. The Shapiro–Wilk Normality test (Prism 8.0, GraphPad Software) was used to assess the normality of the data. Normally distributed data sets are presented as the means ± standard deviation. Data sets that were not normally distributed are expressed as the median value, and the Wilcoxon test or Kruskal-Wallis test was used to assess the statistical significance.

## Results

### Preparation and characterization of dECM@exo

**Fig. 1:**
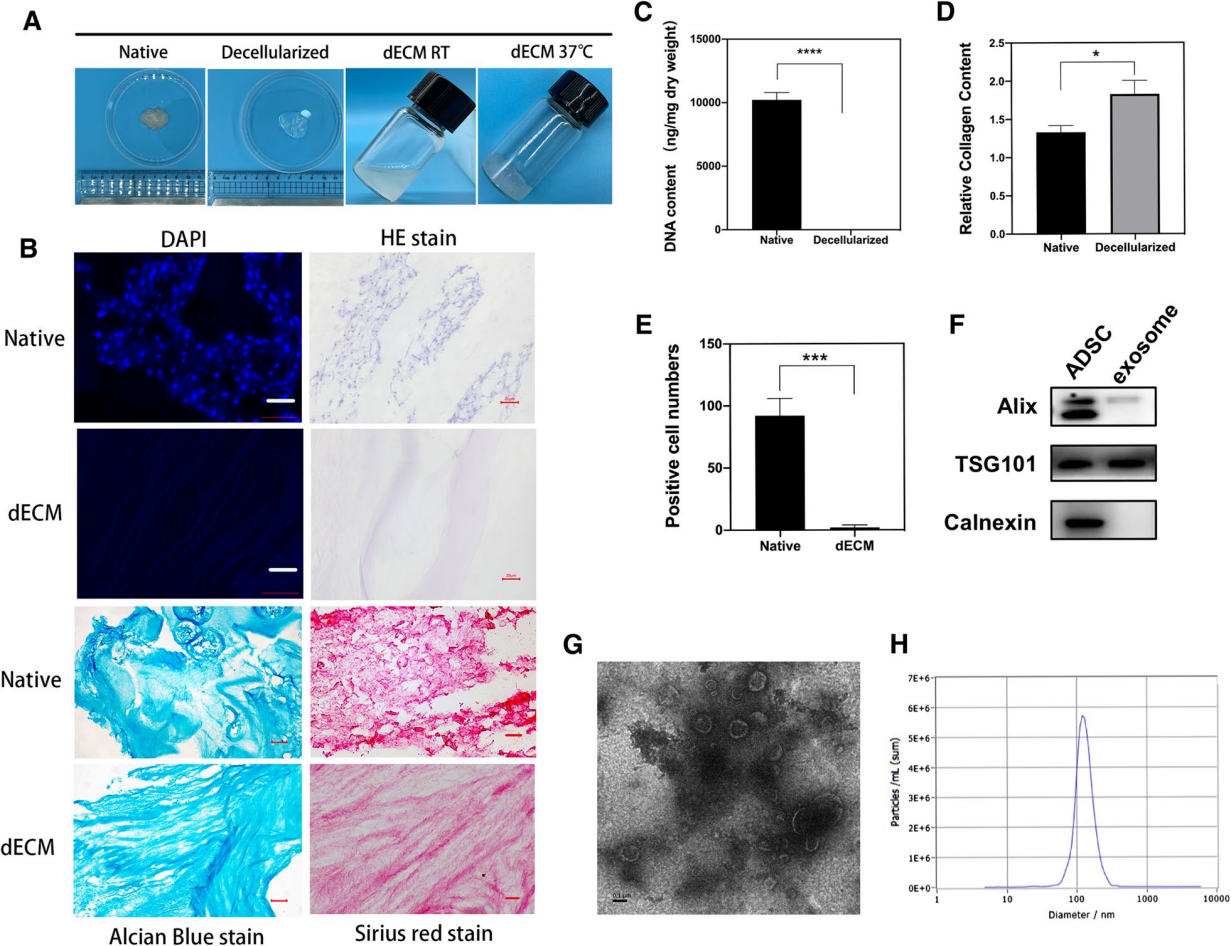
Preparation and identification of dECM and characterization of exosomes. **A** Images of nucleus pulposus tissue decellularization and preparation of hydrogels. **B** DAPI and H&E staining demonstrated decellularization; Alcian blue staining confirmed the retention of glycosaminoglycan; Sirius red staining confirmed retention of collagen fibers. Scale bar = 20 μm. **C** Quantitative determination of DNA content and **D** collagen retention. Data are expressed as the mean ± SD (n = 3). ****P < 0.0001, *P < 0.05. **E** Cell count in the fluorescence field (n = 3). **F** Exosome characterization of Alix, TSG101, and calnexin by Western blotting. **G** TEM analysis of exosomes. Scale bar = 0.1 μm. **H** NTA analysis of exosomes

We fabricated decellularized NP matrix-derived three-dimensional (3D) biocompatible scaffolds, characterized them with ADSC-derived exosomes, and analyzed their biological functions. By testing the DNA content, we found that more than 99% of the DNA in the dECM had been removed. (Fig. 1C). This change was confirmed by DAPI and H&E staining. Alcian blue staining revealed glycosaminoglycan (GAG) retention in the dECM, while Sirius red staining showed collagen structures (Fig. 1B). The collagen content of the dECM was higher than that of the fresh NP tissue (Fig. 1D).

WB showed that the exosomes extracted from the ADSCs contained the characteristic surface marker proteins of exosomes, including Alix and TSG101, and did not express the exosomal negative protein calnexin (Fig. 1F). According to the NTA results, the exosome samples were unimodal in the range of 30–150 nm, which concurs with previous reports (Fig. 1H). Transmission electron microscopy (TEM) analysis revealed that the ADSC-derived exosomes exhibited a cup- or round-shaped morphology (Fig. 1G). Based on these exosome-related results, we can conclude that ADSC-derived exosomes were successfully obtained in this study.

**Fig. 2:**
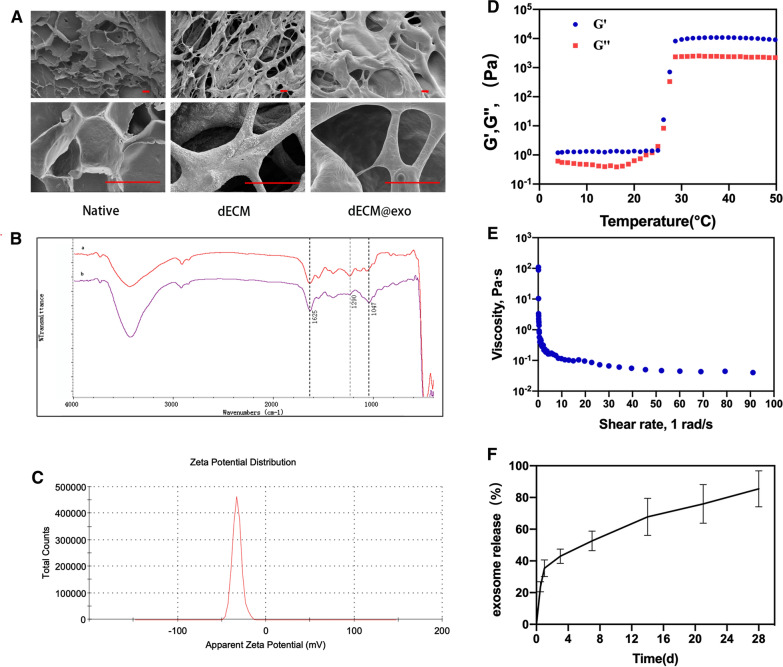
Synthesis and characterization of dECM @ exo. **A** SEM analysis of NP, dECM and dECM@exo. Scale bar = 20 μm. **B** The FTIR of spectra of NP (a) and dECM (b). The absorption band at 1047 cm^−1^ corresponds to the stretching mode of the C–N bond. The peak at 1290 cm^−1^ corresponds to the NH2 groups. The C=O stretching was detected at the wave-length of 1625 cm^−1^. The peak at 1290 cm^−1^ corresponds to the NH2 groups. **C** Zeta potential of exosomes. **D** The G′ and G′′ changes of the dECM@exo hydrogel at 4–50 °C. **E** The viscosity changes of the dECM@exo hydrogel at shear rates of 1 1/s to 100 1/s. **F** Control-release curve of dECM@exo

dECM@exo showed a 3D porous morphology after freeze-drying (Fig. 2A). Curve b exhibited several signals, such C–N (1047 cm^−1^) and NH2 (1290 cm^−1^), which confirmed the presence of many positively charged groups in the dECM (Fig. 2B). Moreover, the zeta potential of the ADSC-derived exosomes was − 32.7 mV, which indicated that the exosomes were firmly bound to the dECM (Fig. 2C).

### Evaluation of the properties of the dECM@exo

The rheological properties of dECM@exo were measured to evaluate their mechanical behavior. At 37 °C, dECM@exo displayed a G′ value that was significantly higher than the G′′ value, suggesting a high viscosity compared with their low viscosity at 4 °C (Fig. 2D). As the shear rate gradually increased, the viscosity of the dECM@exo continuously decreased, indicating that it has good injectable performance (Fig. 2E). The excellent sustained-release ability of the exosomes from the dECM@exo was confirmed by the sustained-release curve. Over a period of 28 days, the dECM@exo progressively released the majority of the exosomes (Fig. 2F).

### Biocompatibility of the dECM@exo in vitro

**Fig. 3:**
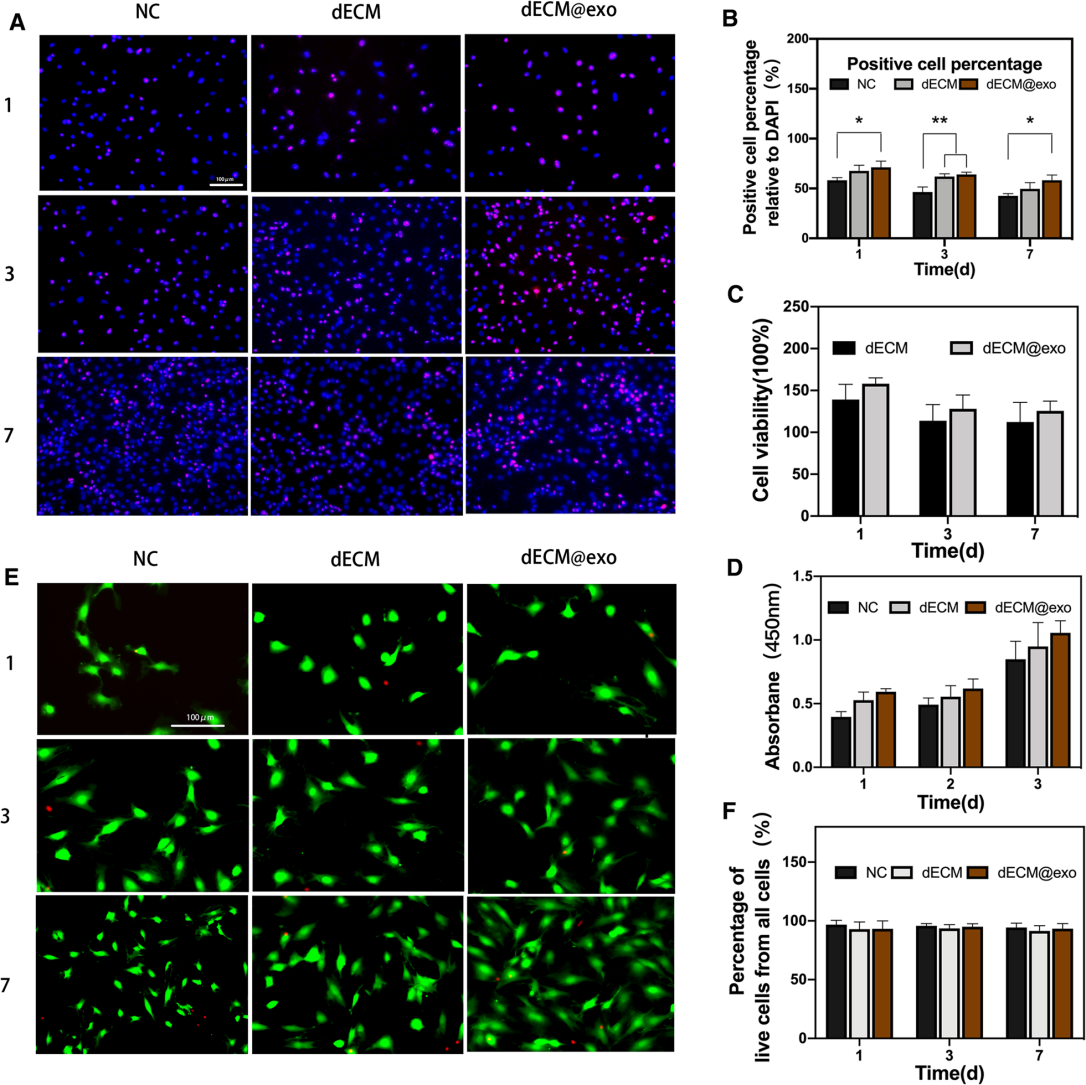
Biocompatibility of dECM@exo. **A** Immunofluorescence microscopy of Ki67 in NPCs. **B** Quantitative detection of the proportion of cells expressing Ki67. **P < 0.01, *P < 0.05. **C** Cell viability of cells cultured with dECM or dECM@exo. **D** The absorbance at 450 nm was compared among the groups. **E** Fluorescence images of living (green) and dead (red) cells in each group. Scale bar = 100 μm. **F** Quantification of the percentage of living cells

As shown in Fig. 3A, the number of NPCs increased with time, while the proportion of proliferating cells decreased. The Ki67-positive ratio of NPCs in the dECM@exo group was higher than that in the NC group (P < 0.05). A CCK-8 assay was performed to detect cell viability, and the results revealed that although the dECM and dECM@exo groups showed a slight increase compared with the NC group, there was no significant difference. As shown in Fig. 3E (with living cells shown in green and dead cells shown in red), nearly all of the cells cultured on the dECM and dECM@exo remained surviving on days 1, 3, and 7. The number of cells increased gradually with the number of days of culture, and the increase was most significant in the dECM@exo group (Fig. 3E).

### The function of dECM@exo in ECM metabolism

**Fig. 4:**
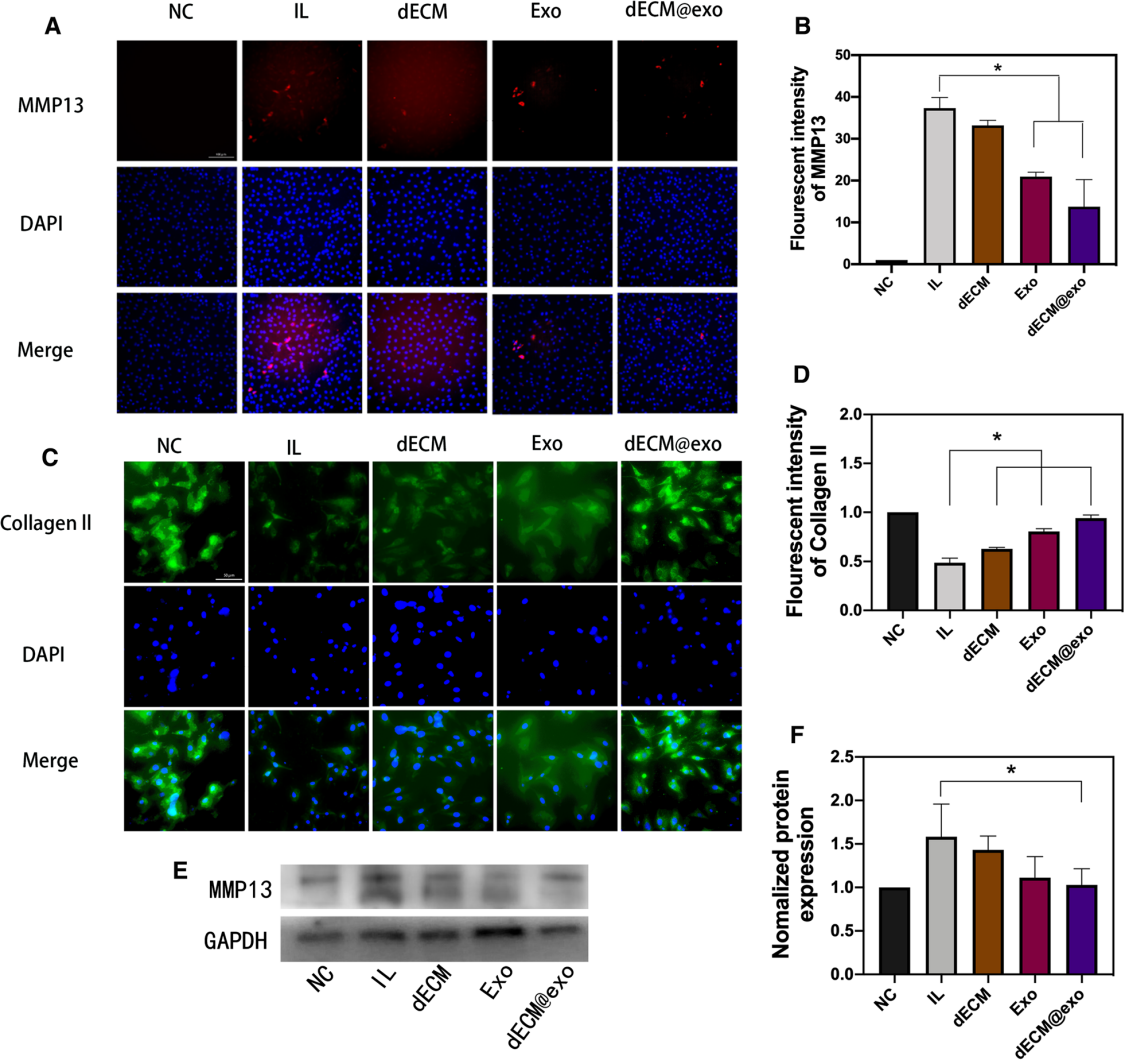
dECM@exo regulates the matrix metabolism of NPCs. **A** Fluorescence images of MMP13 after IL-1β + TNF-α stimulation in the NC, IL, dECM, Exo and dECM@exo groups. Scale bar = 100 μm. **B** Quantitative analysis of the fluorescence intensity of MMP13. Data are the mean ± SD (n = 4). *P < 0.05. **C** Fluorescence images of collagen ll after IL-1β + TNF-α stimulation in each group. Scale bar = 50 μm. **D** Quantitative analysis of the fluorescence intensity of collagen ll in each group. **E**, **F** Western blot of MMP13 in each group. Data are presented as the mean ± SD (n = 3). *P < 0.05

A specific catabolic gene (MMP13) is essential for intervertebral disc degeneration pathogenesis. MMP13 is the major catabolic factor in ECM metabolism. According to the immunofluorescence results, we observed that the fluorescence intensities in the Exo and dECM@exo groups were significantly lower than that of the IL group, and the decrease in the dECM@exo group was more significant (Fig. 4A). These results confirm that exosomes and dECM@exo can downregulate MMP13 and affect the metabolism of the ECM to regulate microenvironmental changes in the early stage of IVDD (Fig. 4B). Moreover, we evaluated the expression of the MMP13 protein by WB, which further confirmed the downregulation effects of the exosomes and dECM@exo on MMP13 (Fig. 4E, F). Finally, according to the type 2 collagen immunofluorescence results, we proved that the dECM@exo downregulation of MMP13 promoted anabolism of the ECM in NPCs, resulting in the accumulation of ECM (Fig. 4C, D).

### dECM@exo inhibit the pyroptosis of NPCs

**Fig. 5:**
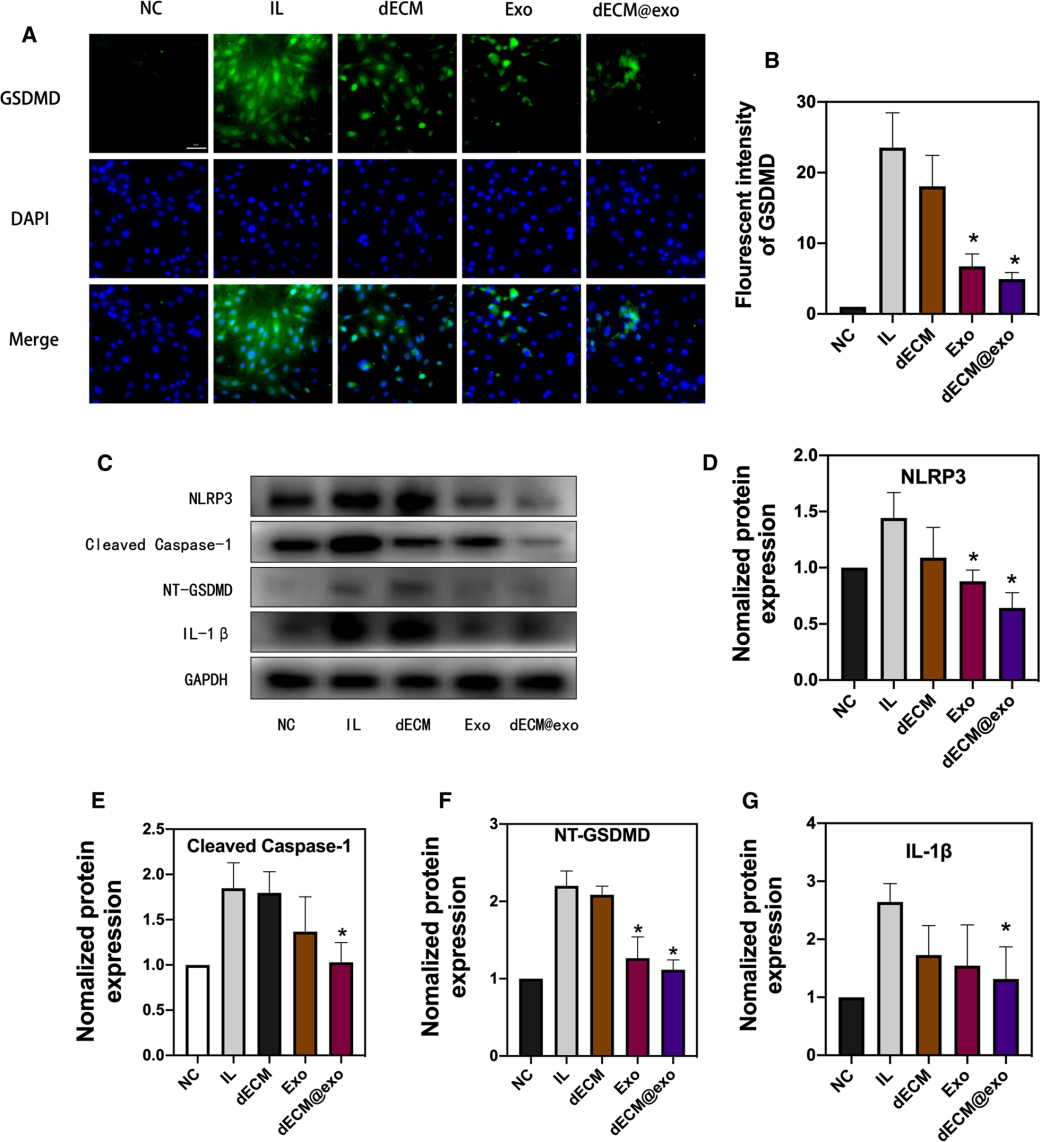
dECM@exo modulated pyroptosis in NPCs. **A** Fluorescence images of GSDMD after IL-1β + TNF-α stimulation in the NC, IL, dECM, Exo and dECM@exo groups. Scale bar = 50 μm. **B** Quantitative analysis of the fluorescence intensity of GSDMD in each group. The data are presented represent as the mean ± SD (n = 4). *P < 0.05. **C** Protein expression levels of NLRP3, cleaved Caspase-1, NT-GSDMD, and IL-1β. **D**–**G** Quantification of protein expression. GAPDH served as a loading control. Data are presented as the mean ± SD (n = 3). *P < 0.05 vs. the IL group

Through the analysis of the GSDMD immunofluorescence analysis, we found that the fluorescence intensities in the dECM@exo and Exo groups were significantly lower than those of the IL group and dECM group, which confirmed that dECM@exo can reduce pyroptosis in NPCs (Fig. 5A, B). Moreover, we assessed pyroptosis in NPCs by measuring the protein levels of NLRP3, cleaved caspase-1, NT-GSDMD, and IL-1β. The IL group and the dECM group showed increased protein levels of NLRP3, cleaved caspase-1, NT-GSDMD, and IL-1β compared with the levels observed in the NC group. Additionally, the dECM@exo and Exo groups showed decreased protein levels (Fig. 5D).

### Imaging evaluation of disc height

**Fig. 6:**
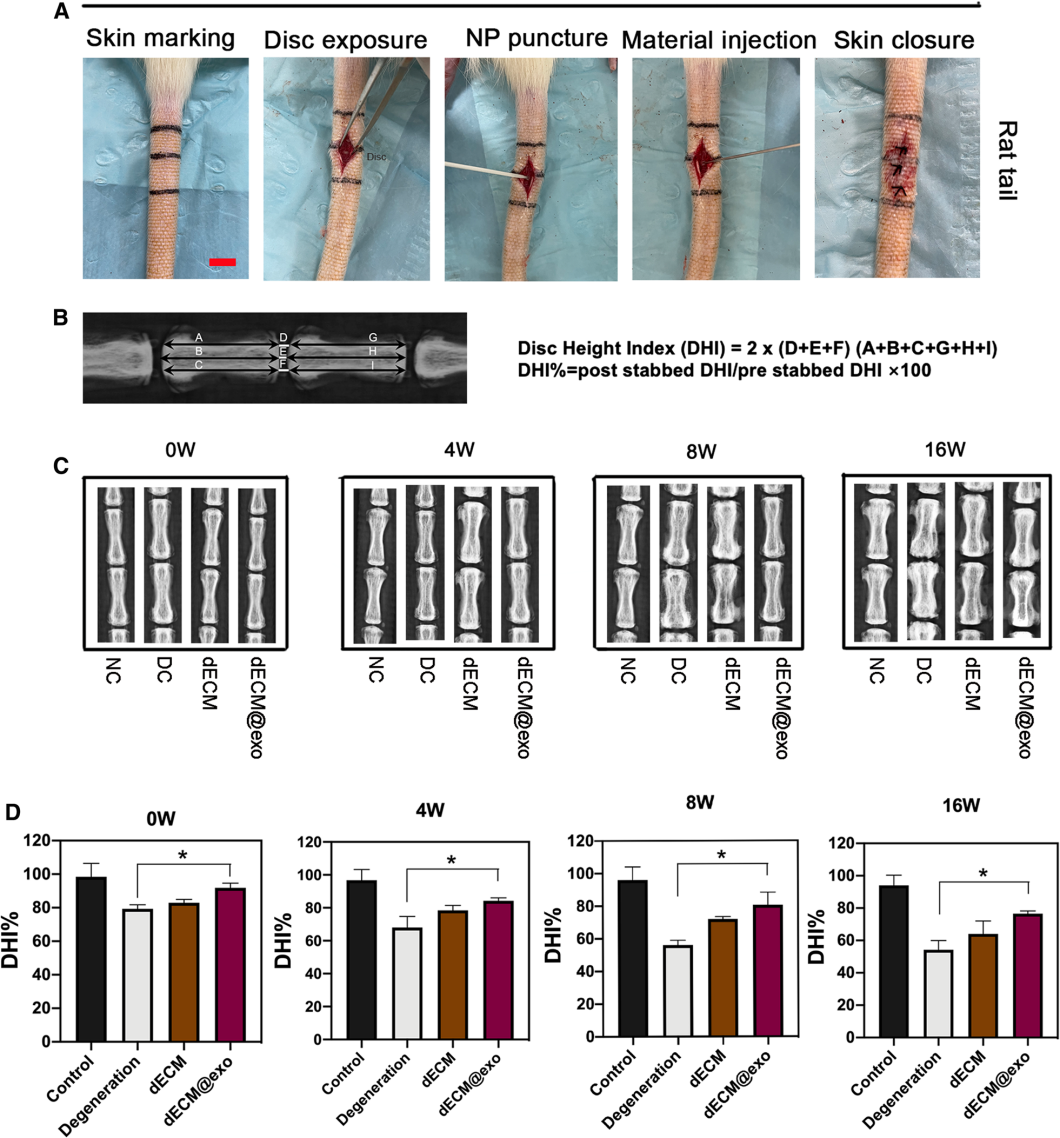
dECM@exo can maintain the mechanical properties of the intervertebral disc. Scale bar = 2 cm. **A** Image of the construction of a disc degeneration model. **B** Calculation method of the disc height index. **C** X-ray images of the intervertebral disc at 0, 4, 8, and 16 weeks. **D** DHI differences among the control, degeneration, DECM and dECM@exo groups at each time period. Data are presented as the mean ± SD (n = 3). *P < 0.05

Based on the X-ray analysis of each group, it can be concluded that the height of the intervertebral disc in each group changed to some extent over time (Fig. 6C). The disc height index (DHI%) values in the other three experimental groups but not the control group decreased conspicuously over time (Fig. 6D). At different time points, the degeneration group showed a significant reduction in DHI%, and the dECM group also showed different degrees of reduction. In contrast, the dECM@exo group showed some disc compression but still maintained a good disc height compared to that observed in the degeneration and dECM groups.

### Histological and immunohistochemical analyses of the IVDs

**Fig. 7:**
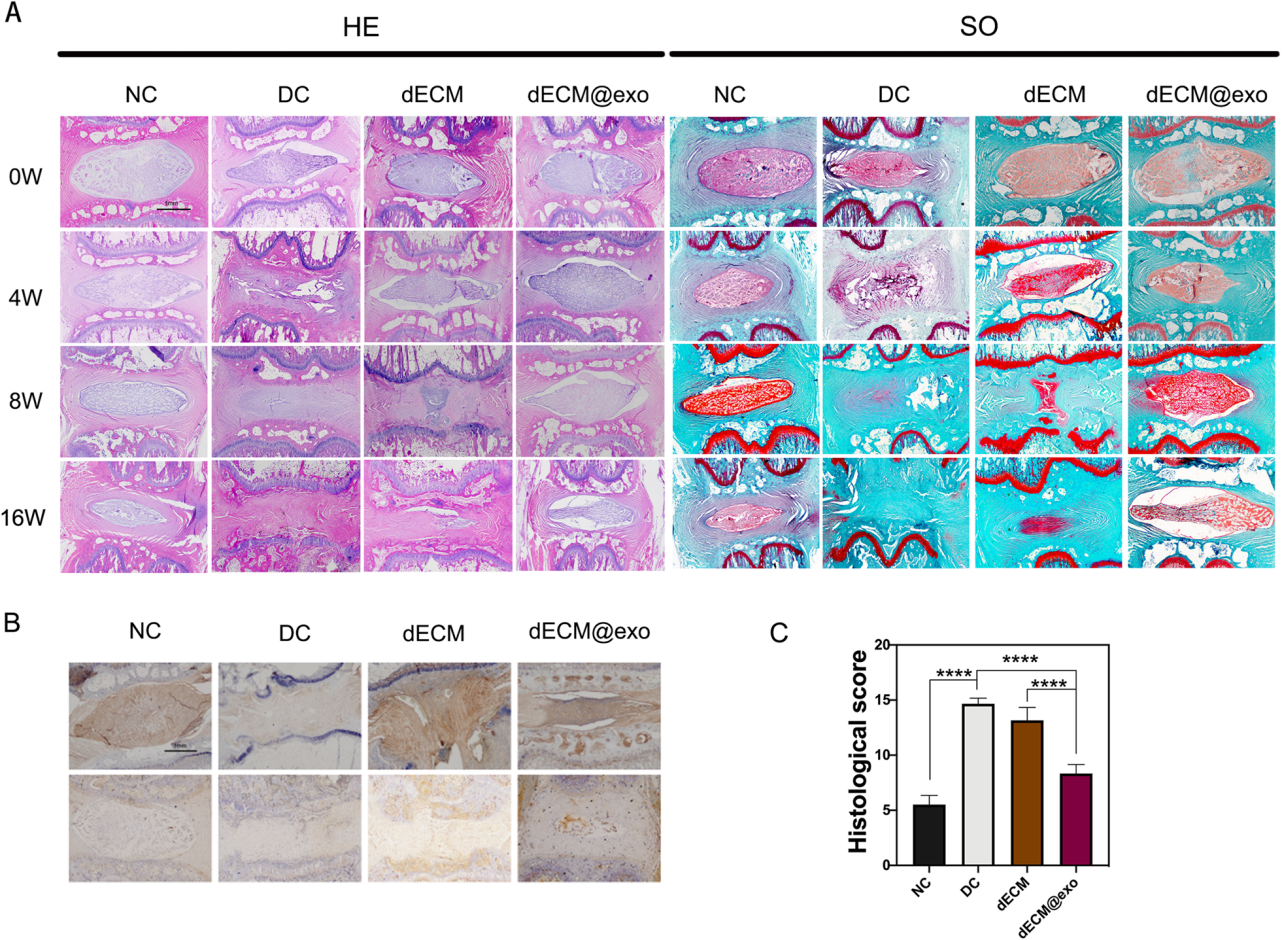
dECM@exo prevented the degeneration of IVDs. **A** Images of H&E and safranin O staining from each group. Scale bar = 1 mm. **B** Immunohistochemical detection of aggrecan and collagen II. **C** Histological grade was evaluated at 16 weeks. Data are the mean ± SD; *P < 0.05

According to the H&E and S-O staining results, we concluded that in the NC group maintained complete IVD morphology (intact NP tissue and well-organized AF) was maintained without degeneration over time; however, the DC group showed significant degeneration over time and finally achieved complete fusion were observed in the DC group. In the dECM group, the disc retained its integrity at the early stage, but with time, only a small amount of NP tissue remained. The dECM@exo group exhibited a more complete IVD structure and morphology than the DC and dECM groups (Fig. 7A). Through immunohistochemistry analysis, it can be concluded that the expression levels of aggrecan and collagen ll in the dECM@exo group were higher than those in the DC and dECM groups, which confirmed the ability of dECM@exo to improve IVDD (Fig. 7B). Histological scores were assigned at week 16, as shown in Fig. 7C. The DC group showed the highest score among all groups, indicating the most serious degeneration, while the score of dECM@exo was only higher than that of the NC group, indicating a less severe degree of degeneration.

## Discussion

In this study, we produced a temperature-sensitive injectable ECM-exosome composite hydrogel that regulates the intervertebral disc microenvironment for the treatment of intervertebral disc degeneration. Compared with traditional biological scaffolds, dECM@exo provides better efficacy through the synergistic effect of ECM and exosomes and is more suitable for the disc microenvironment (Additional file 1: Table S1) [11, 12, 31–36]. ADSC-derived exosomes can slow down the catabolism of ECM by reducing the activity of MMPs. On the one hand, by slowing down the catabolism of ECM, the metabolic disorder of ECM in intervertebral disc degeneration can be corrected so that NPCs can survive. On the other hand, decomposition of dECM is slowed down, allowing exosomes to remain in the disc for up to 28 days. The storage of exosomes by dECM also enables exosomes to inactivate the NLRP3 inflammasome and inhibit the release of inflammatory factors. Thus, inflammatory factors can be inhibited to influence the survival of NPCs. ECM and exosomes act together on NPCs to enhance their tolerance to microenvironmental changes in the early stage of degeneration, reduce their apoptosis, and promote their proliferation to a certain extent.

dECM@exo showed good biocompatibility and did not cause immune rejection. The minimum criterion for successful decellularization is a dry weight DNA content of less than 50 ng/mg, a DNA fragment length of less than 200 bp or the absence of visible nuclear material in DAPI or H&E staining [37, 38]. According to the test results, almost all cell-related components in the dECM were removed, thereby avoiding the elicitation of an immune response by the dECM. The porcine NP matrix has been proven to have regenerative effects in recent studies [39–41]. It can replace the matrix loss caused by the disorder of matrix metabolism and offset the negative effects of metalloproteinases. Additionally, the dECM preserves the original structure while enriching the ECM, enhances structural support while promoting the proliferation of NPCs and resists the effects of inflammatory factors [42]. Most importantly, dECM@exos can effectively slow the release of exosomes while showing a high load rate of exosomes.

There were many positively charged groups in the ECM, and their existence was confirmed by FTIR spectroscopy. By measuring the zeta potential of the ADSC-derived exosomes, we confirmed that they carried a negative charge. This indicates that the dECM can effectively combine with ADSC-derived exosomes through electrostatic attraction, promoting exosome loading [43]. Other mechanisms also mechanisms that allow exosomes to bind to the dECM. Exosomes possess integrin on their cell membrane; as a result, they have the ability to adhere to the ECM as cells through the reaction between integrin and laminin [43–47]. By comparing the dECM and laminin FTIR spectra, it was found that laminin exists in the dECM. However, more objective evidence to confirm this result is needed, as is further research.

Another advantage of using dECM to load exosomes is the special delivery pattern of exosomes in the ECM. Recent studies have shown that matrix stress relaxation allows EVs to overcome confinement, while a higher cross-linking density fluctuates the transport motion through the polymer mesh, resulting in free diffusion and rapid transport. In vitro, the low modulus of the dECM was conducive to the distribution of exosomes [48]. After injection, the modulus of the dECM was higher, and exosomes were able to be fixed in the cages of the dECM. Subsequently, due to their high degree of cross-linking and the presence of surface channel proteins, exosomes can be delivered in the dECM and act on NPCs. Later, as NPCs grow and proliferate in the dECM, they can also serve as channels for intercellular vesicle transport. If dECM@exos loaded with engineered exosomes are used according to this characteristic, targeted drug delivery to NPCs can be achieved, which improves the drug effect of small molecules.

Acellular scaffolds and exosome therapy are two emerging directions of development in the field of disc degeneration therapy. The combination of the two enables them to further enhance their abilities to promote the proliferation and growth of NPCs, as well as their ability to regulate ECM metabolism. According to our in vivo results, their combination can effectively inhibit disc degeneration in contrast to the weak therapeutic effects of IVDD induced by injection of ECM or exosomes alone. Both ECM and exosomes have very low immunogenicity compared to cell therapy, which is an advantage for the clinical application of dECM@exo. For the selection of materials, we chose biological substances with a low chance of body rejection, and we reduced cell rejection to achieve biocompatibility. ADSCs come from a wide range of sources and cause negligible damage to people if they are obtained from the body. The wide range of cellular sources means that exosomes are easy to obtain. Additionally, we used a highly efficient ECM enrichment method, which reduced the required time to 1 day compared in contrast to that in previous studies. Finally, we aim to further develop exosome therapy for clinical use.

## Conclusions

In our study, an injectable enriched ECM biological hydrogels, dECM@exo, was developed to modulate the microenvironment of IVDD. The results demonstrated the biosafety of dECM@exo and its ability to regulate inflammatory complexes and metalloproteinases. As a result, dECM@exo provides a novel strategy for the development of biological therapies for IVDD.

## Supplementary Information

**Additional file 1: Table S1.** Comparison of dECM@exo with ECM scaffolds and exosomedelivery materials in IVDD.

## Data Availability

All data generated or analysed during this study are included in this published article.

## References

[1] Hoy D, March L, Brooks P, Blyth F, Woolf A, Bain C, Williams G, Smith E, Vos T, Barendregt J, Murray C, Burstein R, Buchbinder R (2014). The global burden of low back pain: estimates from the Global Burden of Disease 2010 study. Ann Rheum Dis.

[2] Dagenais S, Caro J, Haldeman S (2008). A systematic review of low back pain cost of illness studies in the United States and internationally. Spine J.

[3] Katz JN (2006). Lumbar disc disorders and low-back pain: socioeconomic factors and consequences. J Bone Joint Surg Am.

[4] Binch ALA, Fitzgerald JC, Growney EA, Barry F (2021). Cell-based strategies for IVD repair: clinical progress and translational obstacles. Nat Rev Rheumatol.

[5] Le Maitre CL, Pockert A, Buttle DJ, Freemont AJ, Hoyland JA (2007). Matrix synthesis and degradation in human intervertebral disc degeneration. Biochem Soc Trans.

[6] Le Maitre CL, Freemont AJ, Hoyland JA (2006). Human disc degeneration is associated with increased MMP 7 expression. Biotech Histochem.

[7] Le Maitre CL, Freemont AJ, Hoyland JA (2004). Localization of degradative enzymes and their inhibitors in the degenerate human intervertebral disc. J Pathol.

[8] Buckwalter JA (1995). Aging and degeneration of the human intervertebral disc. Spine (Phila Pa 1976).

[9] Chao-Yang G, Peng C, Hai-Hong Z (2021). Roles of NLRP3 inflammasome in intervertebral disc degeneration. Osteoarthr Cartil.

[10] Hejazi F, Ebrahimi V, Asgary M, Piryaei A, Fridoni MJ, Kermani AA, Zare F, Abdollahifar MA (2021). Improved healing of critical-size femoral defect in osteoporosis rat models using 3D elastin/polycaprolactone/nHA scaffold in combination with mesenchymal stem cells. J Mater Sci Mater Med.

[11] Alzhavan O, Ghaderi E, Shahsavar M (2013). Graphene nanogrids for selective and fast osteogenic differentiation of human mesenchymal stem cells. Carbon.

[12] Kang S, Park JB, Lee TJ, Ryu S, Bhang SH, La WG, Noh MK, Hong BH, Kim BS (2015). Covalent conjugation of mechanically stiff graphene oxide flakes to three-dimensional collagen scaffolds for osteogenic differentiation of human mesenchymal stem cells. Carbon.

[13] Maldonado-Lasunción I, Haggerty AE, Okuda A, Mihara T, de la Oliva N, Verhaagen J, Oudega M (2021). The effect of inflammatory priming on the therapeutic potential of mesenchymal stromal cells for spinal cord repair. Cells.

[14] Sakai D, Andersson GB (2015). Stem cell therapy for intervertebral disc regeneration: obstacles and solutions. Nat Rev Rheumatol.

[15] Lu K, Li HY, Yang K, Wu JL, Cai XW, Zhou Y, Li CQ (2017). Exosomes as potential alternatives to stem cell therapy for intervertebral disc degeneration: in-vitro study on exosomes in interaction of nucleus pulposus cells and bone marrow mesenchymal stem cells. Stem Cell Res Ther.

[16] Zhang X, Cai Z, Wu M, Huangfu X, Li J, Liu X (2021). Adipose stem cell-derived exosomes recover impaired matrix metabolism of torn human rotator cuff tendons by maintaining tissue homeostasis. Am J Sports Med.

[17] Shi Y, Wang Y, Li Q, Liu K, Hou J, Shao C, Wang Y (2018). Immunoregulatory mechanisms of mesenchymal stem and stromal cells in inflammatory diseases. Nat Rev Nephrol.

[18] Zhang ZG, Buller B, Chopp M (2019). Exosomes—beyond stem cells for restorative therapy in stroke and neurological injury. Nat Rev Neurol.

[19] Cabral J, Ryan AE, Griffin MD, Ritter T (2018). Extracellular vesicles as modulators of wound healing. Adv Drug Deliv Rev.

[20] Xiang H, Su W, Wu X, Chen W, Cong W, Yang S, Liu C, Qiu C, Yang SY, Wang Y, Zhang G, Guo Z, Xing D, Chen B (2020). Exosomes derived from human urine-derived stem cells inhibit intervertebral disc degeneration by ameliorating endoplasmic reticulum stress. Oxid Med Cell Longev.

[21] Hu ZL, Li HY, Chang X, Li YY, Liu CH, Gao XX, Zhai Y, Chen YX, Li CQ (2020). Exosomes derived from stem cells as an emerging therapeutic strategy for intervertebral disc degeneration. World J Stem Cells.

[22] Li Q, Yu H, Sun M, Yang P, Hu X, Ao Y, Cheng J (2021). The tissue origin effect of extracellular vesicles on cartilage and bone regeneration. Acta Biomater.

[23] Mercuri JJ, Patnaik S, Dion G, Gill SS, Liao J, Simionescu DT (2013). Regenerative potential of decellularized porcine nucleus pulposus hydrogel scaffolds: stem cell differentiation, matrix remodeling, and biocompatibility studies. Tissue Eng Part A.

[24] Chan LK, Leung VY, Tam V, Lu WW, Sze KY, Cheung KM (2013). Decellularized bovine intervertebral disc as a natural scaffold for xenogenic cell studies. Acta Biomater.

[25] Fiordalisi M, Silva AJ, Barbosa M, Gonçalves R, Caldeira J (2020). Decellularized scaffolds for intervertebral disc regeneration. Trends Biotechnol.

[26] Naba A, Clauser KR, Hynes RO (2015). Enrichment of extracellular matrix proteins from tissues and digestion into peptides for mass spectrometry analysis. J Vis Exp.

[27] Théry C, Amigorena S, Raposo G, Clayton A (2006). Isolation and characterization of exosomes from cell culture supernatants and biological fluids. Curr Protoc Cell Biol.

[28] Rousseau MA, Ulrich JA, Bass EC, Rodriguez AG, Liu JJ, Lotz JC (2007). Stab incision for inducing intervertebral disc degeneration in the rat. Spine (Phila Pa 1976).

[29] Jeong JH, Jin ES, Min JK, Jeon SR, Park CS, Kim HS, Choi KH (2009). Human mesenchymal stem cells implantation into the degenerated coccygeal disc of the rat. Cytotechnology.

[30] Han B, Zhu K, Li FC, Xiao YX, Feng J, Shi ZL, Lin M, Wang J, Chen QX (2008). A simple disc degeneration model induced by percutaneous needle puncture in the rat tail. Spine (Phila Pa 1976).

[31] Borrelli C, Buckley CT (2020). Injectable disc-derived ECM hydrogel functionalised with chondroitin sulfate for intervertebral disc regeneration. Acta Biomater.

[32] Ligorio C, O’Brien M, Hodson NW, Mironov A, Iliut M, Miller AF, Vijayaraghavan A, Hoyland JA, Saiani A (2021). TGF-β3-loaded graphene oxide - self-assembling peptide hybrid hydrogels as functional 3D scaffolds for the regeneration of the nucleus pulposus. Acta Biomater.

[33] Natarajan A, Sivadas VP, Nair PD (2021). 3D-printed biphasic scaffolds for the simultaneous regeneration of osteochondral tissues. Biomed Mater.

[34] Razavi M, Hu S, Thakor AS (2018). A collagen based cryogel bioscaffold coated with nanostructured polydopamine as a platform for mesenchymal stem cell therapy. J Biomed Mater Res A.

[35] Duan H, Liu Y, Gao Z, Huang W (2021). Recent advances in drug delivery systems for targeting cancer stem cells. Acta Pharm Sin B.

[36] Wu D, Chang X, Tian J, Kang L, Wu Y, Liu J, Wu X, Huang Y, Gao B, Wang H, Qiu G, Wu Z (2021). Bone mesenchymal stem cells stimulation by magnetic nanoparticles and a static magnetic field: release of exosomal miR-1260a improves osteogenesis and angiogenesis. J Nanobiotechnol.

[37] Crapo PM, Gilbert TW, Badylak SF (2011). An overview of tissue and whole organ decellularization processes. Biomaterials.

[38] Gilbert TW, Freund JM, Badylak SF (2009). Quantification of DNA in biologic scaffold materials. J Surg Res.

[39] Kitahara H (1979). Histochemical study of the human intervertebral disc. Nihon Seikeigeka Gakkai Zasshi.

[40] Wachs RA, Hoogenboezem EN, Huda HI, Xin S, Porvasnik SL, Schmidt CE (2017). Creation of an injectable in situ gelling native extracellular matrix for nucleus pulposus tissue engineering. Spine J.

[41] de Vries S, Doeselaar MV, Meij B, Tryfonidou M, Ito K (2019). Notochordal cell matrix as a therapeutic agent for intervertebral disc regeneration. Tissue Eng Part A.

[42] Mwale F, Roughley P, Antoniou J (2004). Distinction between the extracellular matrix of the nucleus pulposus and hyaline cartilage: a requisite for tissue engineering of intervertebral disc. Eur Cell Mater.

[43] Wang C, Wang M, Xu T, Zhang X, Lin C, Gao W, Xu H, Lei B, Mao C (2019). Engineering bioactive self-healing antibacterial exosomes hydrogel for promoting chronic diabetic wound healing and complete skin regeneration. Theranostics.

[44] Chang AC, Uto K, Homma K, Nakanishi J (2021). Viscoelastically tunable substrates elucidate the interface-relaxation-dependent adhesion and assembly behaviors of epithelial cells. Biomaterials.

[45] Sun Z, Yang J, Li H, Wang C, Fletcher C, Li J, Zhan Y, Du L, Wang F, Jiang Y (2021). Progress in the research of nanomaterial-based exosome bioanalysis and exosome-based nanomaterials tumor therapy. Biomaterials.

[46] Chen MB, Lamar JM, Li R, Hynes RO, Kamm RD (2016). Elucidation of the roles of tumor integrin β1 in the extravasation stage of the metastasis cascade. Cancer Res.

[47] Yamada Y, Hozumi K, Katagiri F, Kikkawa Y, Nomizu M (2013). Laminin-111-derived peptide-hyaluronate hydrogels as a synthetic basement membrane. Biomaterials.

[48] Lenzini S, Bargi R, Chung G, Shin JW (2020). Matrix mechanics and water permeation regulate extracellular vesicle transport. Nat Nanotechnol.

