# Supplementary material for: Injectable exosome-functionalized extracellular matrix hydrogel for metabolism balance and pyroptosis regulation in intervertebral disc degeneration
Source: J Nanobiotechnology. 2021 Sep 6;19:264. doi: 10.1186/s12951-021-00991-5 (PMC8419940; doi:10.1186/s12951-021-00991-5)
Supplement: Supplementary file 1 — Additional file 1: Table S1. Comparison of dECM@exo with ECM scaffolds and exosomedelivery materials in IVDD. [file 12951_2021_991_MOESM1_ESM.docx]

Table S1. Comparison of dECM@exo with ECM scaffolds and exosome delivery materials in IVDD.

| Function | Material | Comparison(in IVDD) |
| --- | --- | --- |
| Transplantation of stem cells | Collagen[31] | High production cost |
|  | 3D-printed biphasic scaffolds[33] | Unsuitable mechanical properties |
|  | Collagen + polydopamine[34] | Low action period |
| Differentiation of stem cells | Collagen + graphene[11,12] | Unsuitable mechanical properties |
|  | Decellularized nucleus pulposus[24] | Biological toxicity of crosslinking agents |
| Exosome delivery | Nanocomplex[35] | High clearance of exosomes |
|  | Mesoporous silica nanoparticles[35] | High clearance of exosomes |
|  | Magnetic nanoparticles[36] | Difficult to remove magnetic particles |
